# A new paradigm for classifying and treating HER2‐positive breast cancer

**DOI:** 10.1002/cnr2.1841

**Published:** 2023-05-31

**Authors:** Xuexin He, Sai‐Ching J. Yeung, Francisco J. Esteva

**Affiliations:** ^1^ Department of Medical Oncology Huashan Hospital of Fudan University Shanghai China; ^2^ Division of Internal Medicine MD Anderson Cancer Center Houston Texas USA; ^3^ Division of Hematology/Oncology Northwell Health Cancer Institute at Lenox Hill Hospital New York New York USA

**Keywords:** classification, cure, HER2‐positive breast cancer, paradigm, treatment

## Abstract

**Background:**

Because of the phenomenal success of treatment with monoclonal antibodies and antibody‐drug conjugates targeting human epidermal growth factor receptor 2 (HER2), most patients with early‐stage HER2‐positive breast cancer (HER2+ BC) and some with limited metastatic diseases have been cured, and those who have not been cured have achieved significant improvements in overall survival, which has weakened the role of the TNM staging system in the prognosis of HER2+ BC today. Given that the disease is now highly curable, treatment conception, classification, and modalities should differ from those of cancer types with a poor prognosis. It is warranted to build a new paradigm for classifying and treating HER2+ BC.

**Recent findings:**

In our personal view, we suggest that the classification system should be based not only on traditional anatomy and cancer biology but also on available treatment regimens, their exceptional outcomes, and their toxicities. In this regard, we developed a new concise classification of HER2+ BC based on the TNM staging system, a review of the literature, research results, and our clinical experience, dividing the patients into four distinct groups: curable (lymph‐node negative and small (≤3 cm) early breast cancer), do our best to cure (locally advanced or tumors >3 cm early breast cancer), hope for cure (local‐regional recurrent, limited metastases, and exceptional responders), and incurable (metastatic breast cancer with poor performance status or non‐exceptional responders).

**Conclusion:**

It will assist clinicians in developing an optimal treatment regimen at the outset, curing more HER2+ BC patients and improving their quality of life.

## 1 INTRODUCTION

Breast cancer is the most frequently diagnosed cancer in the world.^1^ Early diagnosis has become increasingly possible in great part due to large screening campaigns and the widespread use of mammography. Approximately 15–20% of breast cancers exhibit HER2/*neu* gene amplification or otherwise overexpress human epidermal growth factor receptor 2 (HER2).^2^ Therapies targeting HER2 (e.g., monoclonal antibodies, antibody‐drug conjugates, and small molecules) have been phenomenally successful.^3^, ^4^ In the two decades since trastuzumab treatment was introduced, most patients with early‐stage HER2‐positive (HER2+) breast cancer have been cured, and those not cured have achieved considerable and long‐lasting improvement in quality of life and overall survival.^2^, ^5^ Furthermore, patients with HER2+ limited metastatic (less extensive / small‐volume burden of metastatic disease) or extensive loco‐regional recurrent disease, or who had metastatic disease but were exceptional responders (achieving a long‐lasting complete response), had a cure rate of more than 30%.^6^, ^7^

The 8th American Joint Commission of Cancer (AJCC) for breast cancer staging system added genomic assays, HER2, hormone receptors, and histologic grade to the classic TNM staging system, to define the prognostic stage grouping.^8^ However, the successful development of HER2 targeted therapies has weakened the weight of tumor size and axillary lymph node involvement on the prognosis of HER2+ breast cancer in recent years. For example, integration of HER2 targeted monoclonal antibodies, antibody‐drug conjugates, and cytotoxic drugs has significantly improved prognosis for patients with HER2+ breast cancer regardless of initial tumor size and lymph node involvement.^9^

In selected solid tumors (e.g., colorectal cancer and non‐small‐cell lung cancer), current AJCC staging criteria takes into account location and extent of distant metastatic disease to provide more accurate prognostic estimates.^8^ This is not the case in metastatic breast cancer, despite wide variations in progression‐free survival rates and overall survival rates across metastatic cancer patients with different subgroups.^10^ This is an area of increasing clinical relevance, particularly in metastatic HER2+ breast cancer where the overall survival rates have improved dramatically over the past two decades.^5^

Because early‐stage HER2+ breast cancer is highly curable with available therapeutic modalities,^2^, ^5^, ^7^, ^11^ the staging (or more accurately, classification) and treatment planning should differ from those cancer types with poor prognosis (e.g., pancreatic cancer, triple‐negative breast cancer). We believe it is necessary to develop a new paradigm to define distinct prognostic subgroups, establish goals of treatment for individual patients and improve the cure rate for HER2+ breast cancer overall.

## 2 CLASSIFICATION AND TREATMENT OF HER2+ BREAST CANCER

**FIGURE 1:**
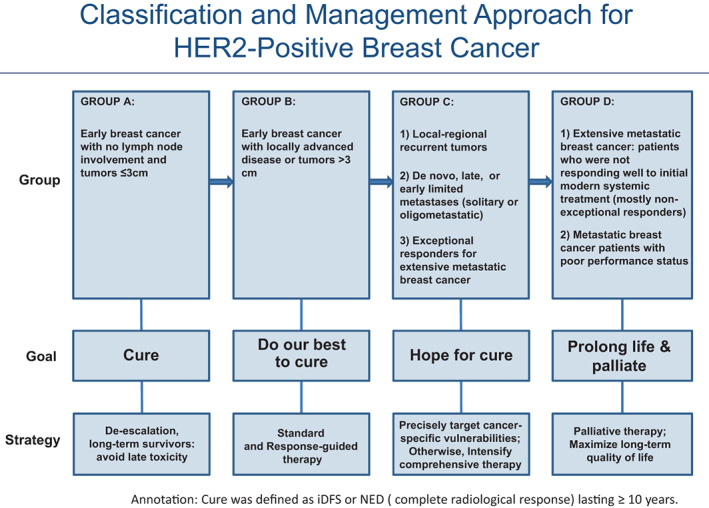
Classification and management approach for HER2+ breast cancer.

The classification system that we propose is based not only on traditional anatomy, clinical characteristics, and cancer biology, but also on available treatment regimens and outcomes (e.g., long‐term complete response, high cure rate), toxicities, and tolerability. In this regard, we offer an update of the recommended current practices for classifying and treating HER2+ breast cancer and associated impediments (Figure 1).

We propose a new classification of HER2+ breast cancer that takes into account clinical, pathological and therapeutic considerations. In our opinion, HER2+ breast cancer be categorized into four distinct groups:

- **Early breast cancer with small (≤3 cm) tumors and no lymph node involvement (Group A).** Approximately 60% of patients with breast cancer belong to this group,^2^ and the cure rate is more than 95%.^11^ Standard adjuvant systemic therapy includes de‐escalation approaches (e.g., 12 cycles of weekly paclitaxel plus 1 year of trastuzumab) that limit the risk of late toxicities in long‐term survivors.^11^, ^12^ For those with large (size >20 mm) or moderate‐sized (20 mm ≥ size≥8 mm) hormone receptor‐negative breast cancer in this group, neoadjuvant therapy may be an option.^13^ Low‐risk patients (e.g., grade I, strongly ER/PR‐positive, low Ki‐67, tumor size <8 mm) may be considered for chemotherapy‐free targeted therapy trials, especially patients with homogeneous HER2 overexpression.^14^
- **Early breast cancer that is locally advanced or has tumors >3 cm in size (Group B).** Patients in this group should receive neoadjuvant therapy consisting of trastuzumab, pertuzumab (HP) with several possible chemotherapy backbones (e.g., AC → THP, THP → AC, TCHP regimens, or trastuzumab‐deruxtecan→THP regimen in the setting of clinical trial). Patients who achieve pathological complete response (pCR) should complete 1 year of HP therapy. Patients with residual disease after neoadjuvant HER2‐targeted therapy should be considered for trastuzumab‐DM1 or novel therapies in the setting of clinical trials.^15^ Patients with radiological complete responses in the axillary lymph nodes should be encouraged to opt for breast‐conserving surgery if medically appropriate (unpublished data). Patients with hormone receptor‐positive disease should receive at least 5 years of adjuvant endocrine therapy after completion of adjuvant chemotherapy. We therefore propose that these patients should be treated with curative intent with standard treatment and response‐guided therapy.
- **Local‐regional recurrent tumors, limited metastases, or exceptional responders to metastatic breast cancer (Group C).** Although individuals with metastatic breast cancer would not be cured according to our oncologic dogma, appropriate treatments have resulted in some of these patients having no evidence of disease (NED) for long periods of time.^5^, ^7^, ^16^ In this setting, the challenge of identifying the optimal potential curable population has emerged, including identifying populations in whom treatments that precisely target cancer‐specific vulnerabilities would be possible or escalation strategies may be beneficial, while avoiding overtreatment in patients with incurable disease. In this proposal, it is suggested that patients in this group have: (1) local or regional recurrence, including contralateral axillary lymph node relapse or sternal metastases, which should be referred to as the treatment principle of locoregional recurrence; (2) de‐novo, late, or early limited metastases, including solitary metastases (e.g., solitary metastases in the brain, bone, or liver) and oligometastases; or (3) exceptional responders achieving a long‐lasting complete response after limited cycles of chemotherapy combined with anti‐HER2 treatment and/or targeted chemotherapy for extensive metastatic breast cancer. Because the patients in this group have hope for a cure but also have high heterogeneity of tumor and host biology, treatments should precisely target cancer‐specific vulnerabilities^17^; otherwise, it is recommended to intensify comprehensive treatment. In line with locally advanced breast cancer regimens, multidisciplinary treatment includes non‐cross‐resistant systemic therapies (e.g., prefer TCHP regimen if initial (neo) adjuvant therapy with AC → THP (or THP → AC), and/or followed by 4–8 cycles of trastuzumab‐deruxtecan), followed by local therapy (e.g., surgery and/or radiation), and optimal consolidation / maintenance treatment according to the response, as long as the patient has good physical / performance status and agrees to the intensive treatment strategies. For indolent oligometastatic diseases, radical surgery or radiation (e.g., stereotactic radiosurgery) without stronger chemotherapy is considered on an individual basis.
- **Extensive metastatic breast cancer (Group D).** The patients in this group are usually considered incurable using conventional therapies but can have long‐lasting survival. Tumors often develop mechanisms of resistance to HER2‐targeted therapy.^18^ Palliative therapy regimens and their impact on quality of life should consider long‐term factors such as extended treatment and toxicity accumulation. After a limited cycle of a single cytotoxic agent with anti‐HER2 treatment, hormone receptor‐positive patients should transition to endocrine therapy. Surgery and radiation therapy can be considered for palliation and long‐lasting survival. For example, patients with pathological fractures (either actual or impending) may benefit from internal fixation. Patients with spinal metastases who require surgery are often considered for separation surgery, followed by stereotactic radiosurgery. Brain metastases become common in later lines of therapy, and many tumors are amenable to stereotactic radiosurgery (e.g., gamma knife, cyber knife) while avoiding whole brain radiation.

## 3 ASSOCIATED IMPEDIMENTS FOR THE CLASSIFICATION AND TREATMENT OF HER2+ BREAST CANCER

Cytotoxic chemotherapy is still an important part of treating HER2+ breast cancer because it kills heterogeneous clones of cancer cells. This is even more important now that we have antibody‐drug conjugates that target HER2. Several chemotherapy agents have been shown to be synergistic with anti‐HER2 agents, especially targeted HER2 monoclonal antibodies. More aggressive management with chemotherapy may be warranted when we have a breast cancer patient with small volume oligometastatic disease. The development of antibody‐drug conjugates, such as trastuzumab‐emtansine and trastuzumab‐deruxtecan, is a big step forward in the treatment of HER2+ breast cancer. In the future, this could mean less use of non‐targeted chemotherapy.^19^

Although immunotherapies inhibiting programmed cell death protein 1 or ligand 1 were not successful for HER2+ breast cancer patients,^20^ antibody‐dependent cell‐mediated cytotoxicity from trastuzumab, pertuzumab, and antibody drug conjugates has played a key curative role by eliminating microscopic metastases, increasing immune surveillance, and preventing late recurrence.^21^ The peptide vaccine GM2 combined with trastuzumab and novel immune‐stimulating antibody drug conjugates may have potential as effective immunotherapies in the future.^22^ Lastly, an immunologically active breast cancer microenvironment (e.g., expression of CD8^+^ tumor infiltrating lymphocytes) has a favorable prognosis for HER2+ breast cancer patients.^23^ Late recurrence after early breast cancer (typically occurring more than 5 years after initial diagnosis and treatment) is a significant clinical issue, especially in hormone receptor‐positive disease.^24^ Late metastatic recurrence arises from residual cancer cells that are reactivated from dormancy, and there is a lack of specific/efficient therapeutic targeting strategies aimed at minimal residual disease (dormant disseminated tumor cells or micro‐metastasis) and dormant metastatic niches.^25^ Advanced liquid biopsy diagnostic methodologies (e.g., circulating tumor DNA detection and analysis) would be integrated into the recognition, molecular characterization, and surveillance of minimal residual disease.^26^

Molecular imaging (e.g., new antibody‐conjugated nuclear probes) and liquid biopsy are promising noninvasive tools for breast cancer screening, guiding and evaluating treatment, and exploring drug resistance mechanisms.^27^ These approaches may promote the development of individualized therapies with high efficacy and low toxicity. The clinical validation of novel imaging modalities and biomarkers that can detect limited metastatic breast cancer is an area of active investigation. Circulating tumor cells and circulating tumor DNA can identify patients with metastatic disease earlier than waiting for symptoms, and help to distinguish oligometastatic from early phase of widely metastatic disease. Clinical trials are needed to determine whether changes in treatment will have an impact on progression‐free survival rates and overall survival rates.

Circulating tumor DNA analysis can also test whether we can use minimal residual disease and exceptional responders to determine which patients can stop treatment for HER2+ breast cancer, whereas HER2‐targeted antibody‐conjugated nuclear probes could be used to guide anti‐HER2 therapy for metastatic brain lesions. De‐escalation of therapy and molecular imaging‐adapted approaches may be able to reduce the risk of late toxicities that continue to plague many long‐term survivors of early‐stage breast cancer.

For HER2+ breast cancer patients with a high cure rate (Group A), comprehensive cancer treatment, cardiovascular complications, and comorbidity management are active areas of investigation and should be paramount clinically, as should nutrition and physical activity for survivors.^28^ In breast cancer survivors, the risk for cardiovascular disease is heightened by cancer treatments (e.g., targeted therapy, chemotherapy, and radiation therapy) and modifiable factors, including diabetes, obesity, physical inactivity, poor diet, and tobacco use.^29^ These risk factors for cardiovascular disease should be addressed promptly. For example, patients with uncontrolled hypertension should avoid anthracycline usage, and type 2 diabetes patients with HER2+ breast cancer are advised to take metformin if not contraindicated.^30^, ^31^

Risk‐adapted and response‐guided therapy is the frontier for tailoring therapy for HER2+ breast cancer.^32^ Among HER2+ early breast cancer patients who had residual invasive cancer after completion of neoadjuvant therapy, the risk of recurrence of invasive breast cancer or death was 50% lower with adjuvant trastuzumab‐emtansine compared to trastuzumab alone.^15^ Additionally, the response‐adapted phase 2 PHERGain trial, in which patients received trastuzumab and pertuzumab using ^18^F‐fluorodeoxyglucose‐PET, experienced a 37.9% complete pathologic response rate compared with the historical rate, suggesting that PET scans can identify responders who may not need chemotherapy or anti‐HER2 treatment.^33^ HER2+ breast cancer patients with de novo presentation, extensive immune infiltration, a small burden of disease and strong HER2 overexpression are more likely to experience long‐lasting responses^34^; these patients are today precluded from curative approaches owing to the presence of distant metastatic dissemination. However, given the substantial improvements in efficacy of new HER2‐directed agents, we believe curative attempts should be investigated.^35^ In our opinion, the definition of cure in HER2+ metastatic breast cancer is long‐term complete radiological remission lasting for at least 10 years, especially for hormone receptor‐positive patients. Achieving a complete response represents a key predictor of long‐term outcomes.^36^, ^37^ Because Group C is a heterogeneous population with complex cancer biology and tumor microenvironment, it is better to precisely target cancer‐specific vulnerabilities; otherwise, it is recommended to intensify treatment with extreme caution in order to maximize the chances of a complete response through the use of novel, highly active anti‐HER2 agents or combination therapy of effective drugs.^16^, ^17^, ^38^

## 4 CONCLUSIONS

The phenomenal success of modern systemic treatment for HER2+ breast cancer has challenged traditional staging systems that were largely based on tumor size, node involvement and presence or absence of overt metastatic disease. Improvements in long‐term survival rates and aiming for cure is not limited to patients with early‐stage breast cancer, pushing the boundaries to include breast cancer with limited metastases and exceptional responders with extensive metastases. For cancer types with a high cure rate (Group A), it is important to limit long‐term toxicities for survivors when we initially design a treatment regimen. We strive to cure locally advanced breast cancer with standard and response‐guided therapy (Group B). We also do our best to precisely target cancer‐specific vulnerabilities for patients with limited metastatic cancer who have hope for a cure (Group C). For patients with incurable cancer (Group D), it is recommended to monitor compliance with therapy and the toxicities of long‐term treatment. It is also recommended to push extensive metastatic HER2+ breast cancer patients who achieved an exceptional response from treatment into Group C, which is a novel response‐guided dynamic classification.

Nowadays, most patients with HER2+ breast cancer can be cured. For patients with incurable disease, our goal is to prolong life and preserve long‐term quality of life. We plan to further validate our classification system in collaboration with international tumor registries. Although further evaluation and refinement will undoubtedly be necessary, this proposal could serve as a solid foundation for future work and potentially serve as an example for other cancer types (e.g., nasopharyngeal carcinoma, prostate cancer) with a high cure rate.

### PREVIOUS PRESENTATION

The content of this manuscript has never been presented anywhere, including in part, or in any format.

### DISCLAIMERS

There are no sponsors for this work.

## AUTHOR CONTRIBUTIONS

**Xuexin He:** Conceptualization (lead); project administration (lead); supervision (lead); validation (lead); visualization (equal); writing – original draft (lead); writing – review and editing (equal). **Sai‐Ching J. Yeung:** Conceptualization (equal); supervision (equal); validation (equal); visualization (equal); writing – original draft (supporting); writing – review and editing (equal). **Francisco Esteva:** Conceptualization (supporting); project administration (equal); validation (equal); visualization (supporting); writing – review and editing (equal).

## CONFLICT OF INTEREST STATEMENT

All authors declare that they have no conflict of interest relevant to this study.

## Data Availability

I confirm that my article contains a Data Availability Statement even if no data is available (list of sample statements) unless my article type does not require one.I confirm that I have included a citation for available data in my references section, unless my article type is exempt.Data sharing not applicable to this article as no datasets were generated or analysed during the current study
